# Rethinking Motor Lateralization: Specialized but Complementary Mechanisms for Motor Control of Each Arm

**DOI:** 10.1371/journal.pone.0058582

**Published:** 2013-03-05

**Authors:** Pratik K. Mutha, Kathleen Y. Haaland, Robert L. Sainburg

**Affiliations:** 1 NM VA Healthcare System, Albuquerque, New Mexico, United States of America; 2 Department of Neurology, University of New Mexico, Albuquerque, New Mexico, United States of America; 3 Department of Psychiatry, University of New Mexico, Albuquerque, New Mexico, United States of America; 4 Department of Kinesiology, Pennsylvania State University, University Park, Pennsylvania, United States of America; 5 Department of Neurology, Pennsylvania State University, University Park, Pennsylvania, United States of America; The University of Western Ontario, Canada

## Abstract

Motor lateralization in humans has primarily been characterized as “handedness”, resulting in the view that one arm-hemisphere system is specialized for all aspects of movement while the other is simply a weaker analogue. We have proposed an alternative view that motor lateralization reflects proficiency of *each* arm for complementary functions that arises from a specialization of *each* hemisphere for distinct movement control mechanisms. However, before this idea of hemispheric specialization can be accepted, it is necessary to precisely identify these distinct, lateralized mechanisms. Here we show in right-handers that dominant arm movements rely on predictive mechanisms that anticipate and account for the dynamic properties of the arm, while the non-dominant arm optimizes positional stability by specifying impedance around equilibrium positions. In a targeted-reaching paradigm, we covertly and occasionally shifted the hand starting location either orthogonal to or collinear with a particular direction of movement. On trials on which the start positions were shifted orthogonally, we did not notice any strong interlimb differences. However, on trials on which start positions were shifted orthogonally, the dominant arm largely maintained the direction and straightness of its trajectory, while the non-dominant arm deviated towards the previously learned goal position, consistent with the hypothesized control specialization of each arm-hemisphere system. These results bring together two competing theories about mechanisms of movement control, and suggest that they coexist in the brain in different hemispheres. These findings also question the traditional view of handedness, because specialized mechanisms for *each* arm-hemisphere system were identified within a group of *right*-handers. It is likely that such hemispheric specialization emerged to accommodate increasing motor complexity during evolution.

## Introduction

The modern view of brain lateralization, built upon early work in patients with unilateral brain damage and more recent work in split-brain patients [1], [2], suggests that each cerebral hemisphere has become specialized for different, but complementary control processes that together govern a particular behavior. Such hemispheric specialization likely emerged to minimize time and energetic costs associated with transmitting information over long distances as brain size grew to accommodate newer functions during evolution. This notion is supported by the observation that an increase in brain volume in primates is accompanied by a decrease in the relative size of the corpus callosum and anterior commissure, and an increase in local intrahemispheric circuitry [3], [4]. Thus, the development of specialized local circuits lateralized to a single hemisphere could allow the emergence of greater behavioral complexity without incurring the cost of always coupling the two hemispheres for every aspect of neural processing [1].

Studies of lateralization of cognitive and perceptual processes have supported the notion that each hemisphere contributes unique mechanisms to the control of a given function. For example, language comprehension recruits the left hemisphere for lexical, semantic and syntactic processing, and the right hemisphere for processing its emotional and non-verbal features such as prosody [5], [6], [7]. Similarly, visual perception is dependent on the synthesis of global features of a stimulus, which occurs largely in the right hemisphere, and characterization of the details of the same stimulus, which occurs primarily in the left hemisphere [8], [9], [10].

Unfortunately, motor lateralization, which may be defined as a difference in motor performance between the two arms, has yet to be appreciated from this perspective. Instead, motor lateralization has primarily been conceptualized in terms of “handedness”, assessed in two ways – first, by noting the preferred arm for doing tasks and second, by examining performance differences between arms on a particular task. Both these approaches have led to a view that one arm (at a population level, often the right arm) is specialized for all aspects of movement, while the other is simply a weaker analogue. Theories postulating the origin of such “lateralization” have ranged from cultural and social influences during development [11], [12] to explaining it as an artifact of left-hemisphere dominance for language [13], [14], [15]. However, not only do these ideas fail to elucidate the mechanisms that give rise to the “superiority” of one arm, they are also not consistent with our understanding of hemispheric lateralization of other complex behaviors such as language or visual perception, which, as stated above, emphasizes the distinct contributions of each hemisphere for optimal behavior.

Prior studies have attempted to describe the mechanistic basis of interlimb performance differences based primarily on the distinction between feedforward and feedback modes of control. For instance, Roy, Elliott and colleagues noted that the dominant arm system demonstrates an advantage in the speed of processing visual and proprioceptive feedback, and proposed a specialization for feedback control for this system [16], [17], [18]. In contrast, the non-dominant system was proposed to better at movement planning or feedforward control based on the finding that reaction times were often faster for the non-dominant arm in healthy humans [19], [20], [21]. However, based on motor deficits seen in unilateral stroke patients, an opposite framework, with a dominant system specialization for feedforward processes and a non-dominant specialization for feedback mechanisms has been proposed [22], [23], [24], [25]. Thus, the feedforward/feedback distinction has not yielded an unequivocal mechanistic explanation for motor lateralization (also see [26], [27], [28]).

We have introduced an alternative hypothesis that motor lateralization is the result of a specialization of each arm-hemisphere system for distinct and complementary motor control mechanisms [29]. According to this view, each brain hemisphere contributes unique control mechanisms to movements of both arms. We posit that motor lateralization emerged to accommodate greater motor complexity during evolution, for example, as tool use and construction became part of the motor repertoire [30], [31]. However, before this idea of hemispheric specialization for movement control mechanisms can be accepted, it is necessary to precisely identify these mechanisms. We have hypothesized that the dominant arm-hemisphere system has become specialized for optimizing dynamic features of movement such as its direction and trajectory shape [32], [33], [34], [35]. Such control is based largely on predictive mechanisms that anticipate and account for the dynamic properties of the arm and the task environment. In contrast, the hemisphere contralateral to the non-dominant arm has become specialized for achieving stable postures by specifying impedance around “equilibrium” positions [36], [37], [38]. For goal-directed arm movements, this control mechanism specifies a “threshold” or “referent” configuration that the arm must achieve [39], [40]. Consistent with this, the non-dominant arm often shows better accuracy and precision in achieving a desired spatial position, particularly when an ongoing movement is perturbed [36], [37]. Interestingly, these two control mechanisms – predictive dynamic control and impedance-based equilibrium point control – have been extensively reported in the literature, with most studies arguing in favor of one or the other for the control of arm movements [41], [42], [43], [44], [45]. In marked contrast, we suggest that these two mechanisms coexist in the brain, and have become lateralized to different brain hemispheres.

Here we provide a clear test of our hypothesis by using a task that directly probes the control mechanisms that result in interlimb differences in motor performance. In this task we occasionally and covertly shifted the starting location of the hand as healthy right-handed adults performed targeted reaching movements in a virtual-reality environment [46]. Two kinds of shifts were implemented – either orthogonal to or collinear with the direction of movement. Of primary interest to us were the “probe” trials on which movements were initiated from the orthogonally shifted start positions, but had the same distance control requirements as veridical (no shift) baseline trials. We predicted that on these trials, the dominant arm predictive control strategy should largely result in the maintenance of movement direction similar to the baseline movements while the non-dominant specialization for position control should result in movement termination at a spatial position that has been learned over multiple trials (i.e. end near the baseline target position). Note that the feedforward/feedback hypothesis makes no prediction about differences in the mean final position of the hand on these probe trials in the current study. Instead, this framework predicts more variable initial directions for the non-dominant arm, and more variable final positions for the dominant arm. We test this idea as well. In contrast, our prior work [46] showed that movements from the collinearly shifted start positions are modified in terms of their extent. Further, we showed that this modification occurs through longer latency corrective changes in motor commands (occurring at least in part during movement deceleration). Thus, these movements are not determined simply by mechanisms that specify either movement direction or a spatial equilibrium position. It is important to emphasize that a condition necessary to observe the predicted movement patterns on probe trials, particularly for the non-dominant arm, is that the specified control strategy does not change relative to baseline conditions. This does not seem to be the case for movements from the collinear start positions [46]. Our hypothesis therefore makes no predictions about movement patterns from the collinearly shifted start positions. Nevertheless, it may be reasonable to consider that non-dominant arm movements will end more consistently near the baseline target while the dominant arm will show better maintenance of movement direction on these trials.

## Materials and Methods

### Ethics Statement

The institutional review board of the New Mexico Veterans Affairs Healthcare System approved the study. All participants gave written informed consent prior to testing according to the principles expressed in the Declaration of Helsinki.

### Participants

Participants were young, healthy, right-handed adults (n = 14, mean age = 23.35 yrs). Handedness was determined using a 10-item version of the Edinburgh inventory [47].

### Experimental Setup

Subjects sat facing a table with their forearm supported over the table using an air sled system. A cursor (diameter = 0.8 cm) representing the position of the index finger tip, a start circle (diameter = 1 cm) and targets (diameter = 2 cm) were projected using a horizontally mounted HDTV onto a mirror placed beneath it. The mirror blocked direct vision of the subjects arm, but reflected the visual display to give the illusion that the display was in the same horizontal plane as the fingertip. Subjects performed reaching movements on the tabletop below the mirror. Position and orientation of the forearm and upper-arm segments were sampled using a Flock of Birds system (Ascension Technology). The positions of the index finger tip, the lateral epicondoyle of the humerus and the acromion were computed using two Flock of Birds markers per arm and recorded using custom software, with the X-Y plane parallel to the tabletop. We used the computed X-Y coordinates of the fingertip to define the projected cursor position. A bib running from the subjects’ neck to the edge of the mirror was used to block the view of the shoulder and upper arm.

### Experimental Task

Each participant performed the task with both the left and the right arm; the starting arm was counterbalanced across subjects. Two blocks of movements were performed with the same arm in succession and then the arm was switched. Each block consisted of 200 movements to a single target located 15 cm from the start circle, either 45 degrees (“Lateral” movement direction) or 135 degrees (“Medial” movement direction) relative to the horizontal. The task was done in a blocked order to ensure the consistency of movements to every target.

#### Baseline trials

The first 40 movements of each block were baseline movements, during which the on-screen cursor position matched the position of the hand. Prior to each trial, the start circle was displayed on the screen and subjects were asked to bring their hand (cursor) into it. After a brief delay, the target for that trial appeared along with an audio-visual “go” signal, which served as the cue for subjects to reach to the target in a single, uncorrected, rapid motion. Cursor feedback was eliminated at this time. Velocity feedback was provided and subjects were encouraged to attain a peak speed of at least 0.5 m/s. One, three or ten point(s) were given based on movement accuracy if this speed requirement was met. Between trials, the cursor was shown only when the index fingertip was within 4.5 cm from the center of the start circle. This was done to prevent subjects from consciously perceiving the altered conditions during probe trials (see below).

#### Probe trials

After the 40 baseline movements at the beginning of each block, we altered the relationship between the hand and cursor position on occasional “probe” trials. On these trials, which were pseudo-randomly interspersed within the remaining baseline trials, the location of the on-screen cursor was displaced from that of the hand. Importantly however, visually, the task remained exactly identical to the baseline trials in that subjects still brought the cursor into the start circle to initiate the trial. However, on these trials, unbeknownst to subjects, their hand was positioned outside the start circle. Post-testing, subjects reported being unaware of this manipulation. Maximum points were awarded on these trials regardless of movement accuracy.

**Figure 1:**
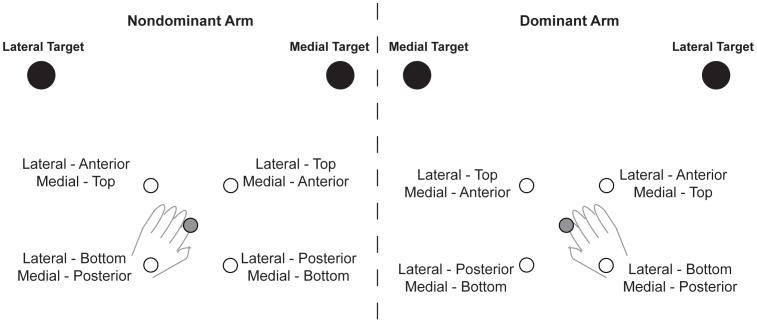
Shifts in start location. Note that shifted start positions were “shared” between the targets. For example, the anterior start position for the lateral movement served as the top start position for the medial movement. The baseline start position was the same for the lateral and medial movement directions.

For each target direction, four different probe start locations were used (Figure 1). These locations were either 4 cm “anterior” or “posterior” to the baseline start position along the target direction (collinear), or 4 cm to the “top” or “bottom” of the baseline start position perpendicular to the target direction (orthogonal). Again, on these trials, visual appearance of the cursor remained the same as baseline, but the starting arm configuration was different. Further, the starting location was changed pseudo-randomly within a block such that no two successive probe trials originated from the same altered start location. There were 8 probe trials per start location for the lateral as well as the medial movement directions, resulting in a total of 32 probe trials per movement direction for each arm. We intentionally limited the number of probe trials to maximize the likelihood that subjects used the same control strategy as they did on the baseline trials. Probe trials were only initiated after the first 40 trials (all baseline) of a block, and a probe trial occurred after 6–7 baseline trials during the remaining 160 trials of that block.

### Data Analysis

All recorded data were low-pass filtered at 12 Hz (third order dual pass Butterworth), and angular kinematic data were differentiated to yield velocity values. The first 20 movements of each block were considered practice and were not analyzed. Probe trials on which subjects failed to move or made extremely curved (almost circular) movements were also excluded after they were identified using outlier Box plots. In total, 8 trials (4 each from the top and bottom start locations) were excluded from a total of 448 probe trials across arms, movement directions and subjects.

We identified movement onset by noting the time of peak velocity and searching backwards in time for the first minimum in velocity below 8% of peak tangential velocity. Movement end was determined by searching forward from peak velocity to find the first minimum below 8% of the peak. Our primary measure of interest was direction error at movement end. For baseline movements, this was defined as the angular difference between the line connecting the center of the start circle and the target, and the line connecting movement start and end points. For the orthogonal probe trials, direction error was calculated relative to a straight line originating from the shifted start location parallel to the baseline movement direction. Counterclockwise direction errors were considered positive, while clockwise errors were considered negative. For each subject, we normalized the direction errors on probe trials by subtracting out the mean baseline direction error. We similarly calculated initial direction errors, defined as the angular difference between the line connecting the start position and the target, and the line connecting the hand locations at movement start and at peak acceleration. For probe trials, these errors were normalized by subtracting the mean initial direction error on baseline movements. We also calculated the position error perpendicular to the baseline target direction. This measure gave us a better estimate of closeness to the baseline target than absolute final position error, which also takes into account the overshoot or undershoot in movement along the target direction. We also used the absolute final position error measure for comparison, and calculated it as the distance between the finger position at movement end and the center of the target. In addition, we computed movement distance as the straight-line distance between movement start and end points. We normalized movement distance for each subject by subtracting the mean extent of baseline movements. For averaging of hand trajectories, the following method was used: first, the X and Y hand-displacement profiles were time normalized, then decimated to 100 points. Then, each series of X and Y displacement profiles were point averaged to yield a mean and SE value for each consecutive point. The mean X and Y values were plotted against each other to yield a mean handpath profile. The SE for X and Y displacements were displayed as horizontal, and vertical error bars respectively.

For statistically comparing the performance of the two arms, we used paired Wilcoxon signed rank tests, pooled across perturbed start positions (top and bottom). For direction errors, absolute values were used during this comparison. Our choice of non-parametric tests was motivated by the fact that the number of probe trials per start location per subject was small (by design) and therefore, the data tended not to be normally distributed.

## Results

### Movements from the Orthogonally Shifted (top and bottom) Start Positions

#### Medial movement direction

**Figure 2:**
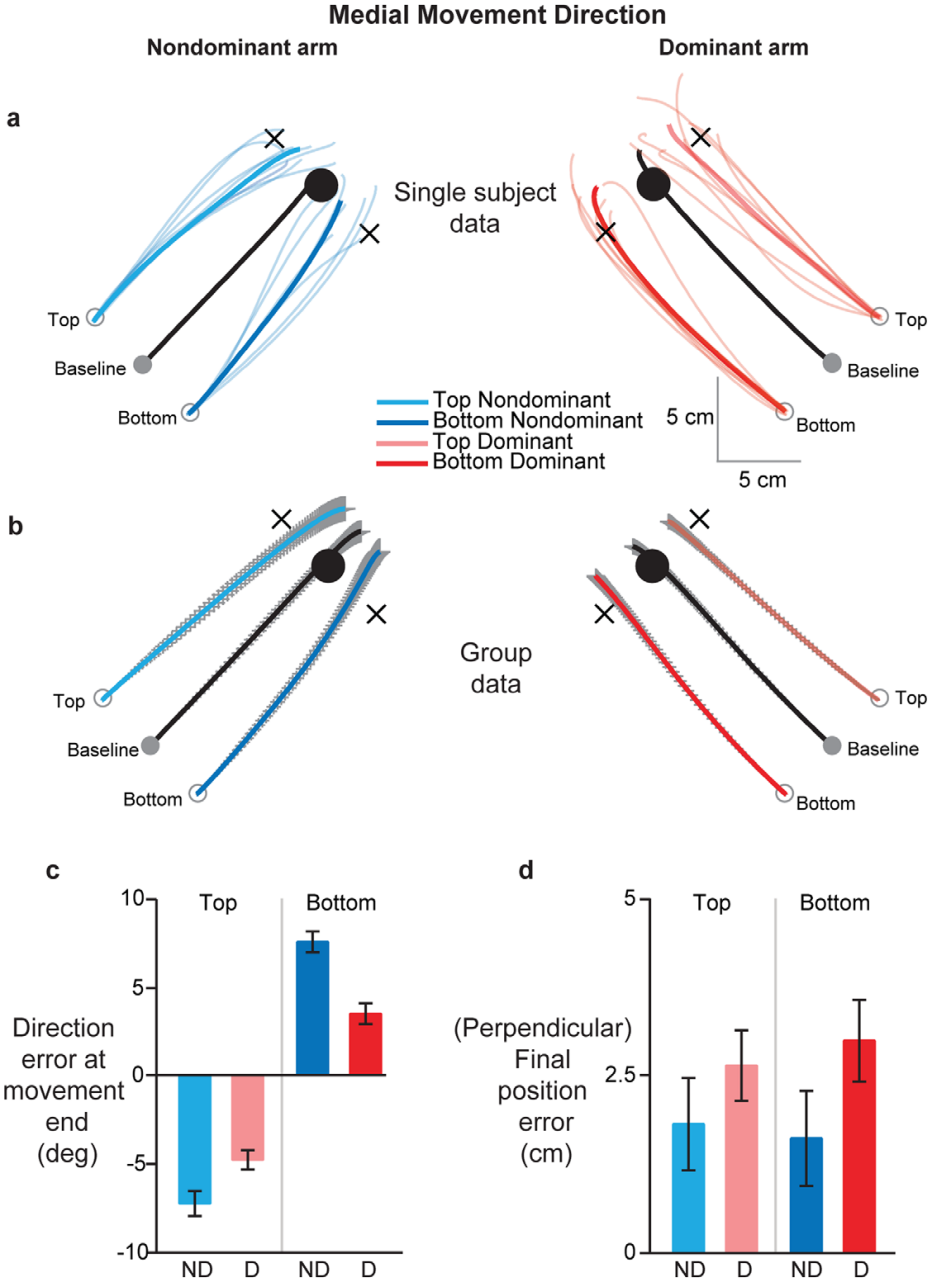
Effects of covertly shifting starting hand position orthogonally on dominant (red) and non-dominant (blue) arm movements in the medial direction. *(A)* Hand trajectories for a single subject. Thin colored lines show individual probe trials, while thick colored lines show the mean of these individual trajectories. Light and dark colors show movements from the top and bottom start positions respectively. Thick black lines represent mean baseline movements. Black crosses represent virtual targets for the shifted start locations, parallel to baseline target direction and at the same distance as the baseline target. Note that subjects saw only the baseline start and target positions. *(B)* Mean hand trajectories across all subjects from the baseline and shifted start locations. Colors and line types represent the same information as in *1A*. Gray lines represent SE. *(C)* Mean direction error at movement end and *(D)* Mean final position error relative to the baseline target across all subjects, separated by start location [top (light colors), bottom (dark colors)] and arm [dominant (red), non-dominant (blue)]. Error bars represent SE.

Figure 2A shows the time-normalized handpaths for the non-dominant (left) and dominant (right) arm for a single subject when moving to a target toward the body midline (“medial” movement direction). Individual probe trials are represented by the thin light colored traces while the mean handpath is shown by a thick trace. As can be seen, the mean baseline trajectories for both arms were directed fairly straight towards the target (thick black lines). On these baseline trials, movement extent was similar across the arms (p = 0.2113), as was final position error (p = 0.4210). The mean dominant arm trajectory from the bottom starting location (thick dark red trace) was essentially identical to the mean baseline movement, but only displaced by the same distance as the start position (4 cm). Similarly, the mean trajectory from the top start position (thick light red trace) ended fairly close to the *virtual* target location (black cross), with only a very slight counterclockwise deviation. Thus, movement direction for the right arm on probe trials was fairly similar to that of baseline trials. In contrast, non-dominant arm trajectories on probe trials deviated much more substantially, and ended much closer to the baseline target. Almost all movements originating from the bottom start location (thin blue lines) were directed counterclockwise relative to a straight line parallel to baseline movement direction. These deviations resulted in the mean non-dominant arm trajectory (thick dark blue trace) ending nearly on the visually displayed baseline target. Similar patterns were observed for the movements starting from the top start position, but with clockwise deviations. This distinct pattern of movements for the dominant and non-dominant arms was consistent across all participants, as revealed by the handpath plots in Figure 2B, which shows the mean time-normalized hand trajectories across all subjects. This figure shows that the dominant, right arm largely maintained the baseline movement direction on probe trials, while the non-dominant arm drifted towards the baseline target position on these trials. However, distance and final position errors tended to be slightly, but statistically significantly, greater in the non-dominant arm compared to the dominant arm (distance: p = 0.0404; final position error: p = 0.0301).

In order to numerically compare the arms in terms of their trajectory differences, we computed the direction of the trajectories at the beginning and end of movement as an angular error relative to a straight line parallel to the baseline target direction, but originating from the perturbed start positions. There were no significant differences between the arms in terms of the mean initial movement direction (p = 0.3596) or its variability (p = 0.1311). However, we found significantly greater direction errors at movement end (Figure 2C) for the non-dominant left arm relative to the right arm (p<0.0001), reflecting the greater tendency of the non-dominant arm trajectories to deviate towards the baseline target. To compare how close to the baseline target the trajectories ended, we computed the final position error perpendicular to baseline target direction. These data are shown in Figure 1D. These position errors were significantly larger for the dominant, right arm than the non-dominant, left arm regardless of starting location (p = 0.0461), but the achieved final positions were not more variable in one arm compared to the other (p = 0.1011). This confirmed that non-dominant arm trajectories ended much closer to the baseline target when starting from the orthogonally shifted start locations.

#### Lateral movement direction

**Figure 3:**
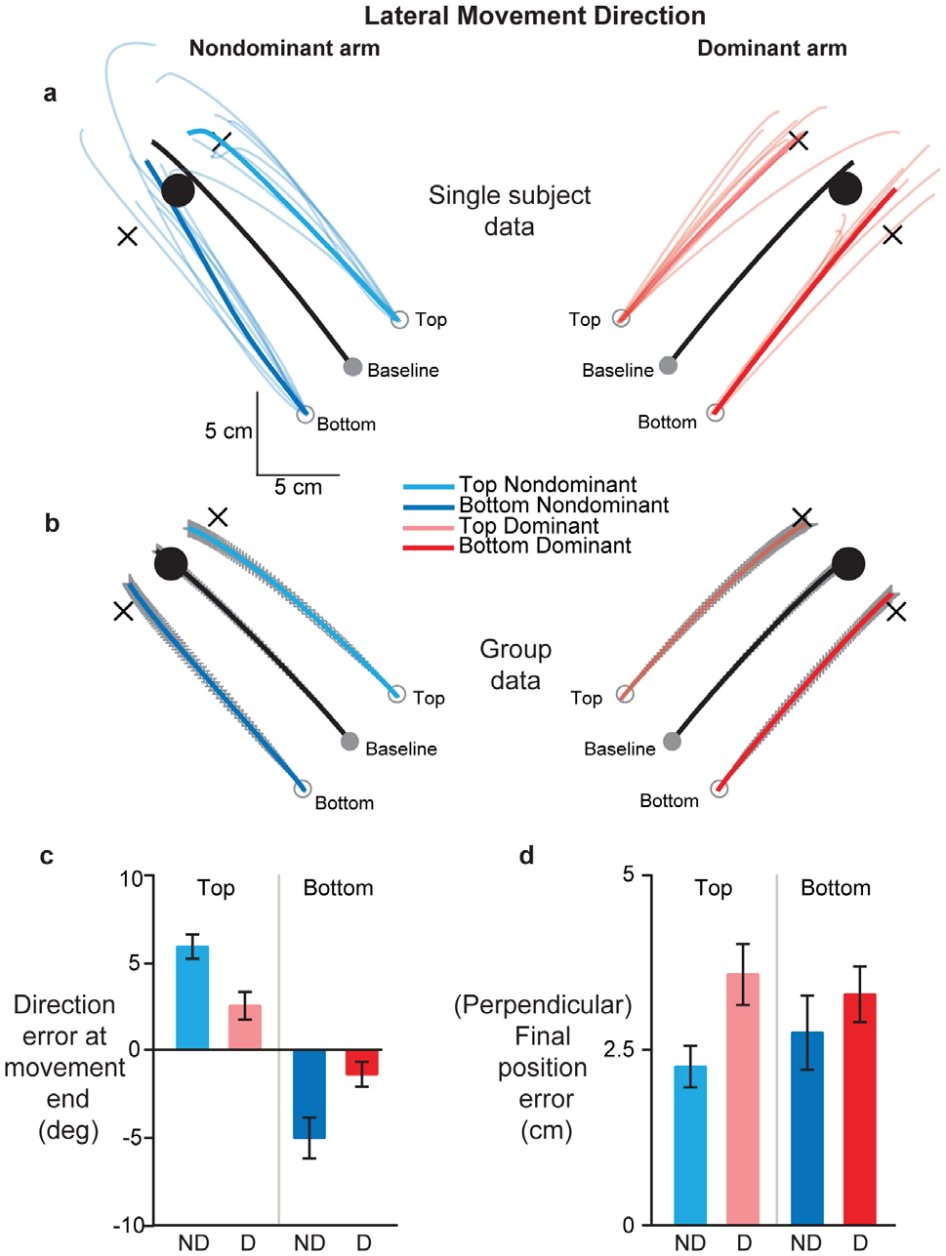
Effects of covertly shifting starting hand position orthogonally on dominant (red) and non-dominant (blue) arm movements in the lateral direction. *(A)* Hand trajectories for a single subject. Thin colored lines show individual probe trials, while thick colored lines show the mean of these individual trajectories. Light and dark colors show movements from the top and bottom start positions respectively. Thick black lines represent mean baseline movements. Black crosses represent virtual targets for the shifted start locations, parallel to baseline target direction and at the same distance as the baseline target. Note that subjects saw only the baseline start and target positions. *(B)* Mean hand trajectories across all subjects from the baseline and shifted start locations. Colors and line types represent the same information as in *2A*. Gray lines represent SE. *(C)* Mean direction error at movement end and *(D)* Mean final position error relative to the baseline target across all subjects, separated by start location [top (light colors), bottom (dark colors)] and arm [dominant (red), non-dominant (blue)]. Error bars represent SE.

A similar pattern of results was observed for movements made away from the body midline (“Lateral” movement direction). The mean baseline handpaths of both arms (thick black lines) for the subject in Figure 3A were straight and directed towards the baseline target. Movement extent (p = 0.3521) and final position errors (p = 0.8341) were not different between the arms on baseline trials. Similar to the medial movement direction, mean dominant arm movements from the bottom (thick dark red trace) and top (thick light red trace) start positions were almost parallel to the baseline trajectory, with only a small counterclockwise deviation noted for the trajectory from the bottom start position. In contrast, non-dominant arm movements showed larger deviations from the straight line connecting the perturbed start locations to their respective virtual targets (blue traces). In fact, the mean non-dominant arm trajectory from the bottom start position ended on the baseline target. This pattern of larger deviations towards the baseline target was observed across all subjects, as shown in the handpaths of Figure 3B. However, no significant interlimb differences were evident for movements from these start positions in terms of distance (p = 0.2369) or final position error (p = 0.3545).

While initial movement direction was not different between the arms (p = 0.3808), its variability was larger in the non-dominant arm (p = 0.0190). However, our critical measure of direction error at movement end, shown in Figure 3C, was significantly larger for the non-dominant arm relative to the dominant right arm (p = 0.0002), and, consistent with this result, position error relative to the baseline target (Figure 3D) was significantly smaller (p = 0.0295) for the non-dominant arm relative to the dominant arm on probe trials. Positional variability at movement end was also higher in the non-dominant arm (p = 0.0218), consistent with the trend seen during the early phases of the movement. Collectively, these findings indicate that non-dominant arm movements tended to diverge inward toward the baseline target, while dominant arm movements were directed straight and parallel to the baseline movements on probe trials.

### Movements from the Collinearly Shifted (Anterior and Posterior) Start Positions

**Figure 4:**
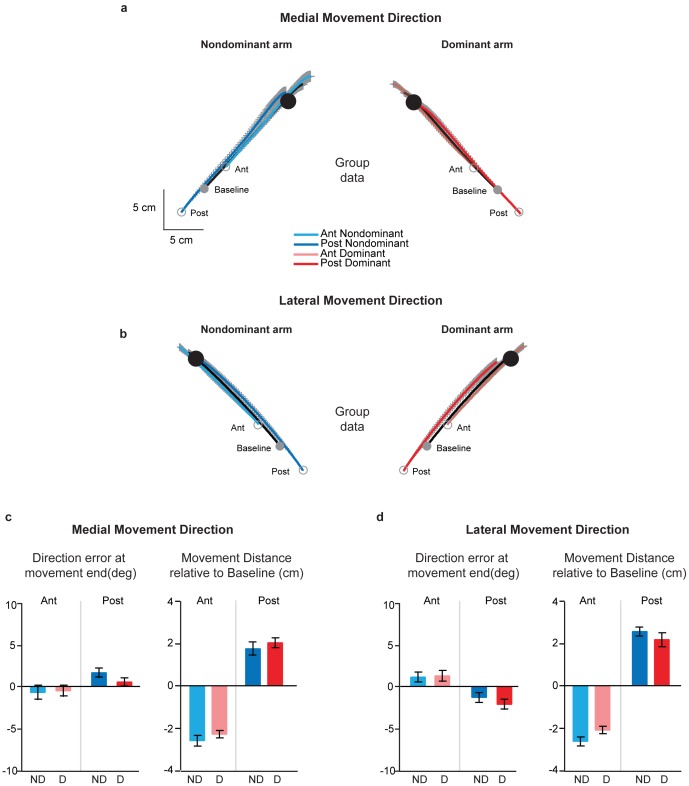
Effects of covertly shifting starting hand position collinearly on dominant (red) and non-dominant (blue) arm movements. *(A)* Mean hand trajectories in the medial direction across all subjects from the baseline and shifted start locations. Light and dark colors show movements from the anterior and posterior start positions respectively. Thick black lines represent mean baseline movements. Gray lines represent SE. Note that subjects saw only the baseline start and target positions. *(B)* Mean hand trajectories in the lateral direction across all subjects from the baseline and shifted start locations. Colors and line types represent the same information as in *4A*. *(C)* Mean direction error at movement end (left panel) and normalized movement distance (right panel) for movements in the medial direction, separated by start location [anterior (light colors), posterior (dark colors)] and arm [dominant (red), non-dominant (blue)] Error bars represent SE. *(D)* Same information as *4C*, except for movements in the lateral direction.

Figures 4A and 4B show the group averaged handpaths for movements starting from the baseline, anterior and posterior start positions made in the medial and lateral directions respectively. In general, regardless of direction, movements from the anterior start positions tended to overshoot the baseline target, while those from the posterior start positions ended fairly close to that target. Importantly however, this trend was not different between the dominant and non-dominant arms. While movements from the anterior and posterior start positions were initiated in the same direction as baseline, notably, movements from the anterior start positions were shorter, while those from the posterior start positions were longer in extent compared to baseline trials (p<0.05 in all cases). There were again no apparent differences between the arms in terms of this tendency to shorten or lengthen movements from the collinearly shifted start positions, regardless of movement direction.

Initial movement direction was not significantly different between the arms for probe trials from the anterior and posterior start positions for either the medial (p = 0.7966) or the lateral (p = 0.1261) movement direction. Further the variability in initial movement direction was also not significantly different between the arms in both the medial (p = 0.2409) and the lateral (p = 0.10) directions. This interlimb similarity in movement direction was maintained at movement end, as shown in the left panels of Figures 4C and 4D. We found no difference between the arms in the direction error at movement end on probe movements made in the medial (p = 0.07) or lateral (p = 0.2664) directions. Thus movements made from the anterior and posterior start positions were similar across the two arms in terms of direction during the early and terminal phases of movement. As stated earlier, there were no apparent differences between the arms in the magnitude of the shortening or lengthening from the anterior and posterior start positions respectively. To confirm this statistically, we compared the distance moved by each arm (normalized to baseline) from these two start locations. This is shown in the right panels of Figures 4C and 4D, in which negative values indicate that movements were shortened, while positive values indicate that movements were of longer extent compared to baseline trials. We found no differences in the (normalized) distance moved between the arms, regardless of movement direction (medial direction: p = 0.2291, lateral direction: p = 0.7901), indicating that both arms equally shortened and lengthened their movements. Collectively, these results indicate no significant interlimb differences on probe trials on which movements were initiated from the collinearly shifted start positions.

## Discussion

This study was motivated by the fact that prior studies have not provided a clear understanding of the neural mechanisms underlying lateralization within the motor system. Several previous studies have quantified performance differences between the two arms [17], [20], [48], [49] but have not directly addressed the mechanisms that might give rise to these differences. Based on our previous results, we have developed a framework of motor lateralization, termed “dynamic dominance” [34], and put forth the idea that dominant arm performance reflects a controller that predicts and optimizes arm and task dynamics, whereas non-dominant arm performance relies on a controller that is specialized for making movements and achieving a stable goal by specifying impedance around “equilibrium” positions. In this study, we implemented covert shifts in the starting location of the hand either collinear with or orthogonal to the direction of hand motion and predicted differences in movement patterns of the two arms based on a preferential reliance on one of these two strategies. While we found no significant interlimb differences in movements initiated from the collinearly shifted start locations, we noted clear differences between the arms for movements initiated from the orthogonally shifted start locations. On these trials, dominant arm movements tended to be straighter and parallel to baseline movements, consistent with a mechanism that predictively accounts for associated changes in arm mechanics to specify the direction and shape of the movement trajectory. In contrast, non-dominant arm movements from these orthogonally shifted positions reflected a mechanism that specifies a final position that has been learned over multiple trials (the baseline target position), i.e. an equilibrium point control strategy. Our results bring together these two long-standing, yet competing theories of movement control, and uncover the possibility that they coexist in the brain, in different hemispheres.

### Lack of Interlimb Differences in Movements from Collinearly Shifted Start Positions

It is important to first discuss the lack of interlimb differences in movements from the anterior and posterior start positions. Movement directions at the beginning and end of motion, as well as the magnitude of shortening and lengthening of movement extent in response to these shifts in start location, were similar between the arms. Our prior work using a similar paradigm [46] showed that movement patterns from collinearly shifted start locations rely to a large extent on feedback mediated control. Thus the control of movement distance relies on mechanisms that are slightly distinct compared to movements driven primarily by mechanisms that specify trajectory features or a spatial goal to be achieved. Note that our hypothesis, particularly for non-dominant arm motion, is that of a control mechanism that is also feedback based, but specifies impedance around specified “equilibrium” positions. How this feedback-mediated, impedance-based control scheme interacts with feedback-based distance control mechanisms remains is not clear. In our case, it is likely that when required to modify movement extent, subjects actually modify their specified equilibrium position online. This precludes the ability to make specific predictions about interlimb differences for modifications to movement extent. It should be noted that we previously demonstrated that interlimb differences in control of movement extent produced differences in torque profiles, but not extent accuracies [46]. This is consistent with our collinearly shifted start position conditions in the current paradigm, which did not lead to differences in extent accuracies between the limbs.

### Interlimb Differences in Movements from Orthogonally Shifted Start Locations

In contrast to movements from the collinear start positions, our results indicated clear differences in movements initiated from the orthogonally shifted start locations. It is important to point out that these findings cannot be explained solely on the basis of a distinction between feedforward or open loop and feedback or closed loop control mechanisms. Applied to our study design, this hypothesis does not make specific predictions regarding the mean final positions of the two arms on probe trials, but suggests larger variability for the non-dominant and dominant arms at the beginning and the end of the movement respectively. We did not find this to be the case. Mean final positions were clearly distinct between the arms on orthogonally perturbed probe trials. In addition, non-dominant arm variability was slightly (but statistically significantly) greater at the beginning of the movement only for movements made in the lateral direction, and contrary to the feedforward/feedback hypothesis, continued to be so even at movement end. Further, variability of movements made in the medial direction was not different between the arms either at the beginning or at the end. Thus, our data suggests a mode of control that appears to be distinct from a general feedforward versus feedback specialization for the dominant and non-dominant arm-hemisphere systems.

We made two key observations in the movement patterns on these probe trials. First, we did not observe any differences in movement direction during the early phases of the movement. Second, we did not observe complete convergence of non-dominant arm trajectories onto the baseline target. The angular deviation was about 60% of that required to land the arm exactly on that target. Similarly, dominant arm movements, particularly in the medial direction, were not completely parallel to baseline trajectories. A recent computational model proposed by Yadav and Sainburg [50] could help explain these findings. This model proposes a serial hybrid control scheme, in which movements of *each* arm are initiated with a predictive control strategy and terminated with a final position controller that uses “impedance” mechanisms to stabilize the arm at a goal position. Thus, while movements of both arms were initiated and terminated using the same strategies, the critical difference between the two arms was characterized by *when* the switch from predictive to positional control occurred. In the current study, the similar movement direction during the early phases of movement might reflect the initial reliance on the feedforward controller to initiate movements of both arms. This initial reliance on predictive control, which drives the arm parallel to the baseline target direction, could also explain why non-dominant arm movements might not completely converge on the baseline target. Similarly, the small deviation at the end of the dominant arm movement might reflect a late switch to impedance-based position control that draws the arm slightly towards the highly practiced baseline target position. In other words, both arms draw on each hemisphere’s control specialization to different extents, resulting in an arm movement that reflects the combined contributions of both hemispheres.

Our framework thus emphasizes bilateral hemispheric contribution to movements of each arm. Such bi-hemispheric control is consistent with observations from previous neuroimaging studies that cortical areas of both brain hemispheres are active during unilateral arm movements [51]. Our studies in stroke patients with unilateral damage to the left or right hemisphere also provide strong support for this view. These patients show similar deficits in both ipsilesional and contralesional arms that depend on the side of damage [33], [52]. For instance, left hemisphere damaged patients show deficits in adapting initial movement direction to novel visuomotor perturbations when moving their contralesional [33] as well as their ipsilesional arms [52], [53]. Interestingly, right hemisphere damage does not adversely impact such adaptation, but often results in poor movement accuracy, which does not occur with left hemisphere damage [35], [52], [53]. These studies have thus shown that both hemispheres cooperate during the performance of unimanual actions in healthy individuals, and damage to one of them likely removes its competition with the intact hemisphere, thereby making the intact hemisphere’s contribution more prominent in movement patterns of either arm. While these studies have been instrumental in demonstrating a critical role for each hemisphere during movements of each arm, our current results make a novel contribution by identifying the control mechanism that each hemisphere contributes to each arm’s motion. It is important to point out however that given the largely crossed neural innervation of our effectors [54], [55], it is the arm contralateral to a hemisphere that primarily shows the signatures of that hemisphere’s motor control specialization. Our current data corroborate this view.

Another important observation in the current study was that non-dominant arm movements in the medial direction tended to be longer than those of the dominant arm on probe trials, but not on baseline trials. One possible explanation for these findings is that the misalignment between the visually and proprioceptively signaled start positions lead to an incorrect estimate of the actual hand position, which, in turn produces errors in movement extent [56]. However, if differences in processing visual-proprioceptive misalignments were the only determinant of the interlimb differences in extent on the probe trials, we would expect movement distance and final position errors to be larger even for the lateral movement direction, which wasn’t the case. Why the non-dominant arm shows greater overshoot in only the medial direciton is not entirely clear. It is likely that mechanisms that control movement extent are influenced by direction dependent variations in interjoint coordination requirements [57], [58], the control of which has been shown to be different for the two arms [49].

### Motor Lateralization as a Result of Hemispheric Specialization for Distinct Motor Control Mechanisms

Prior studies have almost always viewed motor lateralization as “handedness” (either hand/arm preference or greater “skill” in one hand/arm across tasks) rather than arising from specialization for each arm-hemisphere system for different aspects of control within “right-” or “left-handed” individuals. Our findings question this traditional view of handedness, because specialized mechanisms for *each* arm-hemisphere system were identified within a group of *right*-handers. We suggest that the dominant hemisphere provides predictive control mechanisms that specify movement direction and shape, while the non-dominant hemisphere stabilizes the arm at a desired goal position by specifying impedance around that position. A question that arises then is what drove such lateralization during the course of evolution? One possibility is that lateralization was driven by the need to be computationally efficient while accommodating increased complexity in behaviors [59]. Newer functions and increased behavioral complexity could be adapted through an increase in brain size, but this also meant a greater time and energy costs associated with communicating information among processing units. These costs could be avoided by developing modules that support different aspects of a function in different cerebral hemispheres and by developing local intra-hemispheric circuits among frequently communicating modules, i.e. through lateralization. Lateralization may have therefore served an adaptive purpose, leading to its selection during evolution. This explanation also likely accounts for the finding that larger brains (across different species) show a smaller corpus callosum and anterior commissure, and greater intra-hemispheric connectivity [3]. This reasoning also suggests that lateralization of many behaviors, including motor actions, may share a common evolutionary basis. What remains to be explored is how the development of such motor lateralization relates to the emergence of handedness (hand preference) at the population level.
